# Successfully Treated Lung and Renal Metastases from Primary Chondrosarcoma of the Scapula with Radiofrequency Ablation and Surgical Resection

**DOI:** 10.1155/2019/6475356

**Published:** 2019-06-09

**Authors:** Sho Sekito, Manabu Kato, Kouhei Nishikawa, Yuko Yoshio, Masahiro Kanai, Hideki Kanda, Kiminobu Arima, Yoshiki Sugimura

**Affiliations:** ^1^Department of Nephro-Urologic Surgery and Andrology, Mie University Graduate School of Medicine, Tsu, Mie, Japan; ^2^Department of Urology, Suzuka General Hospital, Suzuka, Mie, Japan

## Abstract

Since chondrosarcoma is a relatively rare type of malignant bone tumors characterized by its ability to produce a cartilage matrix and aggressive behavior, a consensus clinical management strategy has not been established. We report a 55-year-old woman who presented with renal metastasis arising from chondrosarcoma of the scapula. Chondrosarcoma of the left scapula was diagnosed 15 years earlier. After surgical resection of a local recurrence in the left scapula, she received focal radiofrequency ablation (RFA). She underwent focal RFA and surgical resection for a total of 21 times for lung metastases. Because invasion of the renal pelvis was suspected from urine cytology, she underwent laparoscopic nephroureterectomy. The histopathological findings showed metastatic chondrosarcoma involving the right renal parenchyma. The patient has remained clinically stable without recurrence for 18 months. To the best of our knowledge, this is the first report of metastatic chondrosarcoma of the lung and renal parenchyma with involvement of the renal pelvis in which remission was achieved with multimodal treatment including RFA and surgical resection.

## 1. Introduction

Chondrosarcoma is the second most common primary malignant bone tumor after osteosarcoma in the United States [1, 2]. Chondrosarcoma shows varying histopathology and clinical behavior consisting of skeletal (central) and extraskeletal (peripheral) types. Among these, conventional central chondrosarcoma is the most frequent type [2]. Based on histopathological features, including cell atypia, mitotic figures, and cellularity, chondrosarcoma is divided into four groups: benign enchondroma and malignant Grade 1, 2, and 3 chondrosarcomas. Chondrosarcoma requires treatment, with surgery being the mainstay of treatment since chemotherapy and radiation therapy have not demonstrated any efficacy in chondrosarcoma patients [1–4]. Surgical procedures have been developed to treat chondrosarcoma, even though the tumors are prevalent in different locations in the body and are of different grades. No medical consensus has been reached on whether wide resection or adequate surgical marginal resection, or even interventional radiology (IVR), should be performed; therefore, clinical studies are necessary to determine the best method of achieving remission in chondrosarcoma patients using a multimodal approach. Chondrosarcoma metastasis to the kidney is relatively rare—some cases have been reported in the literature [5–8]. Here, we present a case of renal metastasis arising from primary chondrosarcoma of the scapula that was treated with multiple procedures, including radiofrequency ablation (RFA) and surgery. A short review of the literature is provided to evaluate the possibility of multimodal treatment resulting in a clinical benefit in metastatic chondrosarcoma.

## 2. Case Report

**Figure 1:**
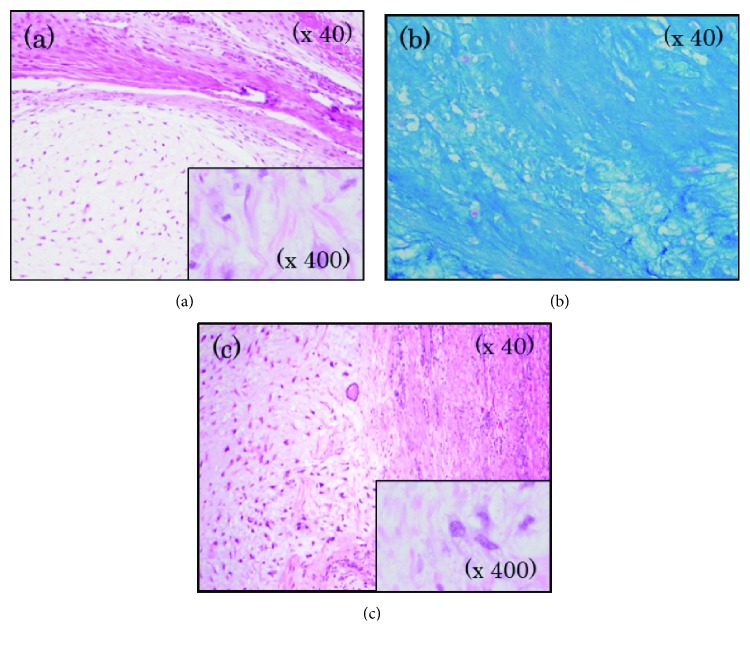
Pathological examination. (a) The primary tumor of her left shoulder demonstrated the sheets of differentiated cartilage cells. (b) Immunohistochemical staining showed positive for toluidine blue reflecting cartilage cells containing acidic mucopolysaccharide. (c) Pathological examination in the kidney was similar to that in the primary tumor of her left shoulder.

A 55-year-old woman who presented with gross hematuria was admitted to our department for a slow-growing right renal mass monitored over time by computed tomography (CT). Chondrosarcoma of the left scapula was diagnosed 15 years earlier. She presented with left shoulder pain, and a left scapula X-ray revealed a 7 cm mass with calcification. Magnetic resonance imaging (MRI) displayed a low signal on T1-weighted and a high signal on T2-weighted. She underwent scapula and humerus wide margin resection of the primary tumor with artificial humeral head replacement. The tumor was composed of cartilage cells growing a lobular architecture with eosinophilic reticulum and myxoid stromal tissue and was diagnosed as Grade 2 chondrosarcoma (Figure 1(a)). Initial surgical treatment showed no evidence of a residual tumor in the disease area and was deemed a successful surgery. Two years after the initial resection, she developed a local recurrence in the left scapula and subsequently underwent extensive resection surgery for both the tumor and clavicle. No residual tumor was detected following surgery. In the following year, multiple bilateral lung nodule shadows appeared on CT. Video-assisted thoracic surgery (VATS) was performed after being diagnosed with metastatic chondrosarcoma to the lung. She proceeded to undergo 13 RFA procedures for lung metastatic chondrosarcoma, with the RFA procedure being performed by an experienced radiologist at our institute. Cooled RFA electrodes (Cool-tip, Valleylab) with a 2 or 3 cm exposed tip were connected to a generator (series CC-1-100, Valleylab) and were inserted into the tumor. Power was applied for a period of 10-12 min using an impedance control algorithm [9]. Six years after RFA treatment, following multiple treatments to the same lesion, VATS was again conducted for a left upper lobe lung metastasis refractory tumor. She then underwent six additional RFA procedures for metastatic chondrosarcoma to bilateral lower lung lobes over the next 5 years. In total, she received 19 RFA and 2 VATS procedures for lung metastases. No relapse of the pulmonary metastasis occurred in 4 years prior to being admitted to our department.

**Figure 2:**
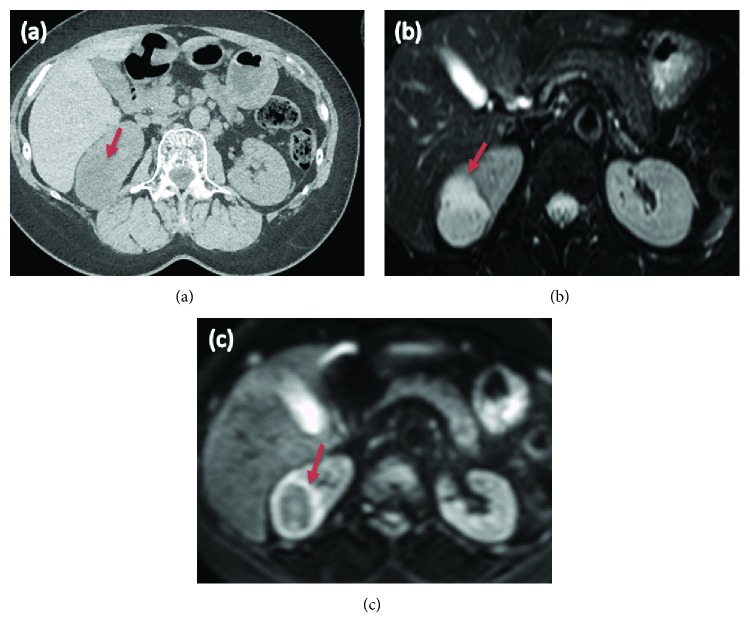
(a) CT scan demonstrated a cyst in the upper half of the right kidney and no septa or calcifications (arrow). (b) T2-weighted MRI showed a cystic tumor with a high signal (arrow). (c) Diffusion-weighted MRI (DW-MRI) showed rim enhancement (arrow).

**Figure 3:**
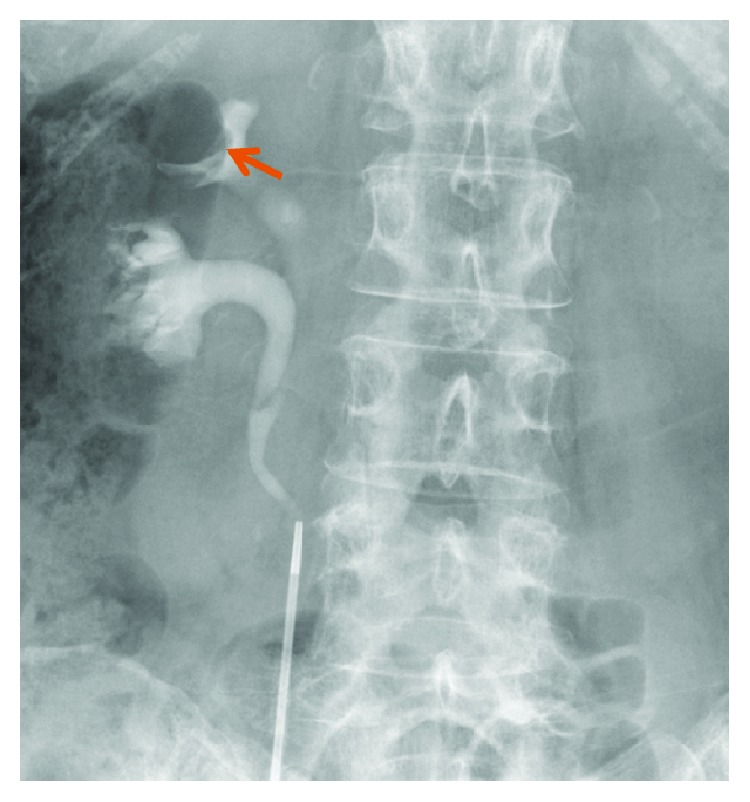
On retrograde pyelography, an incomplete double ureter was showed and a solitary filling defect was observed in the upper pole of the right renal pelvis (arrow).

**Figure 4:**
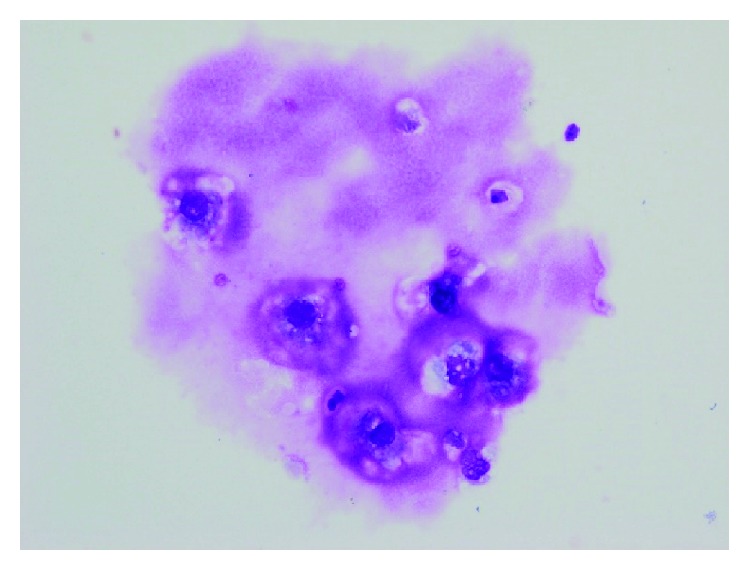
Urine cytology demonstrated low-grade malignant cells with a mucus-like substance (×400).

**Figure 5:**
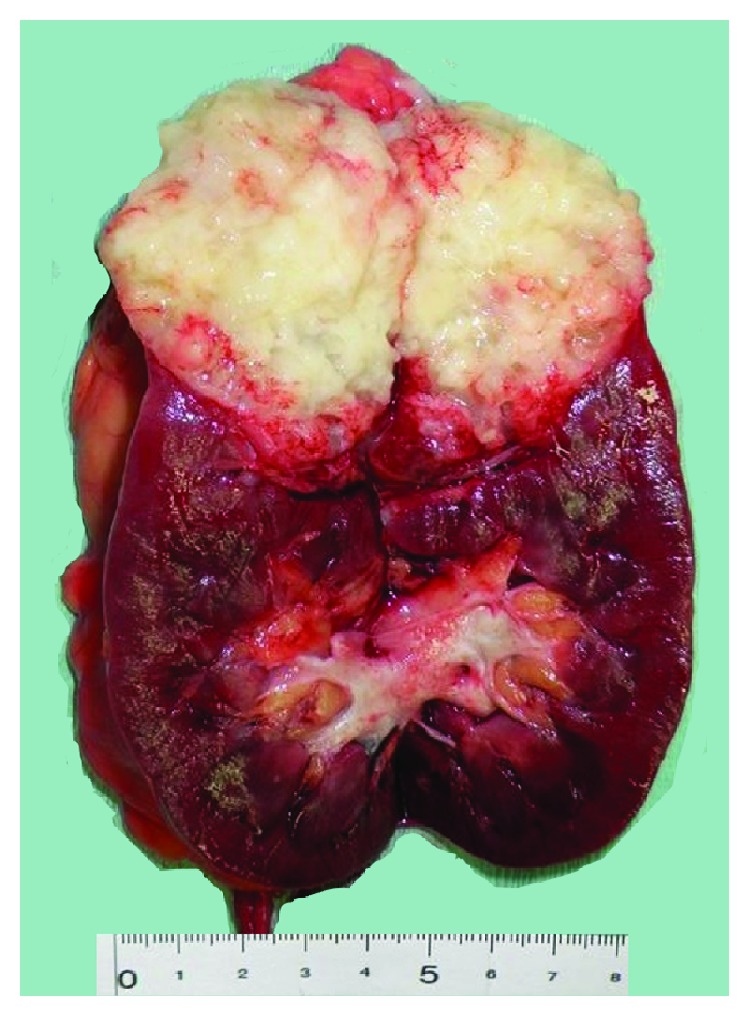
Tumor located in the upper pole with invasion into the renal pelvis.

At the time of admission to the urology department, the patient's laboratory results were within normal ranges, except for the presence of red blood cells in the urinalysis. Three years before the latest admission, CT revealed a cystic tumor in the upper half of the right kidney. Over a period of 3 years, the cyst developed from 14 mm to 46 mm. No septa or calcifications were observed in the tumor (Figure 2(a)). Hydronephrosis was not present, and the right ureter was normal. The patient did not undergo a contrast-enhanced CT due to iodine allergy. A 45 mm cystic tumor without septa, calcifications, or solid components was seen in the upper pole of the right kidney on MRI, displaying a low signal on T1-weighted and a high signal on T2-weighted MRI. Diffusion-weighted (DW) MRI showed rim enhancement. Contrast-enhanced images showed a thick, slightly enhanced cystic wall, and the tumor was classified as a Bosniak IIF cystic renal lesion (Figure 2(b)). Retrograde pyelography demonstrated a solitary filling defect in the upper pole of the right renal pelvis (Figure 3). Urine cytology demonstrated low-grade malignant cells with a light green stained large reticulum and a mucus-like substance (Figure 4). The preoperative differential diagnosis included cystic renal cell carcinoma, carcinoma of the renal pelvis, and metastatic chondrosarcoma. The patient underwent laparoscopic nephroureterectomy, partial cystectomy, and regional lymph node resection. Macroscopically, the resected specimen contained a well-circumscribed tumor measuring 5 × 4 cm. The tumor involved the renal pelvis, and the cut surface of the tumor was white and jelly-like (Figure 5). Histopathological findings showed a myxoid tumor in the tumor wall that invaded the renal pelvis. A cartilaginous tumor composed of atypical cartilage cells was observed. No tumor cells were present in the ureter. Considering the primary tumor pathological findings, we hypothesized that the tumor was composed of cartilage cells with a high cell density and enlarged nuclei (Figure 1(a)). The tumor cells were positive for toluidine blue staining, confirming a cartilaginous component (Figure 1(b)). The kidney tumor was also diagnosed as Grade 2 chondrosarcoma (Figure 1(c)). The resected lymph nodes were negative for metastasis. The final pathological diagnosis was chondrosarcoma metastasis to the kidney. The patient's postoperative course is promising, and she has remained disease-free for over 18 months of follow-up.

## 3. Discussion

Chondrosarcoma is a tumor that originates from cartilage [4]. Compared to osteosarcomas, chondrosarcomas tend to be more prevalent in the older population and exhibit a higher frequency of central locations [10]. Clinical symptoms are nonspecific, with pain being the most frequent symptom; swelling and a palpable soft tissue mass have also been described [1, 11]. Differential diagnoses include juxtacortical chondroma, parosteal osteosarcoma, and periosteal osteosarcoma [2, 11]. Imaging findings of conventional chondrosarcoma typically show a mixed lytic and sclerotic appearance. The sclerotic areas reflect cartilage matrix mineralization [11]. Both intraosseous and extraosseous chondrosarcomas with nonmineralized components typically show low attenuation and mineralization on CT imaging. Contrast-enhanced CT demonstrates a mild peripheral rim and septal enhancement. Higher-grade lesions may show higher CT attenuation, similar to that of a muscle, and more contrast enhancement, reflecting increased cellularity and a reduced water content. The nonmineralized components of chondrosarcoma have high signal intensity on T2-weighted MRI, reflecting the high water content of hyaline cartilage [11, 12]. Therefore, a renal metastasis from chondrosarcoma resembles a complicated renal cyst on MRI. In the present case, the CT and MRI findings were similar to those of images of the local recurrence after the first resection of chondrosarcoma in the scapula. In addition, among primary malignant bone tumors, chondrosarcoma is characterized by a high apparent diffusion coefficient (ADC) on MRI [13]. The combination of CT, contrast-enhanced MRI, DW-MRI, and ADC may increase the diagnostic accuracy of a preoperative imaging workup for patients with renal tumors.

Most chondrosarcomas are slow-growing tumors with low metastatic potential that are considered to be refractory to both chemotherapy and radiation therapy due to a low percentage of actively dividing cells and vascular components [2, 10]. Therefore, for most cases, surgery (wide or en bloc excision) is the only therapeutic option [2, 14]. According to the National Cancer Database report, the overall 5-year survival rate is 75.2% [15]. Angelini et al. reported 5-year survival rates of 99%, 92%, and 77% for patients with Grade 1, 2, and 3 conventional chondrosarcomas, respectively [10]. Metastatic disease develops in 24% of patients, with a 5-year survival rate of 18% [16]. Chondrosarcomas primarily metastasize to the lung; however, other metastatic sites include the humerus, femur, sternum, brain, liver, pleura, heart, and ureter. Tumors recur in 18% of patients, with a mean period between primary surgery and relapse of 24 months [16]. Italiano and colleagues reported that patients with metastatic advanced chondrosarcoma treated with anthracycline-based chemotherapy experienced mean overall survival of 18 months [17]. Radiotherapy might provide some benefits in patients with conventional chondrosarcoma, although its role may be palliative for symptom relief [2]. As shown in these previous reports, neither radiotherapy nor chemotherapy appeared to be curative. However, there is one report of a child with high-grade chondrosarcoma in the base of the skull that was successfully treated with chemoradiation [18]. Thus, in addition to surgery, multimodal therapy including chemoradiation may control chondrosarcoma for some time. The present case experienced a long disease-free period after treatment with RFA, suggesting that a well-planned procedure for local or distant disease can mitigate high-grade chondrosarcoma.

A review of literature identified nine case reports of primary chondrosarcoma of the kidney and three case reports of renal metastasis from chondrosarcoma [5–8] (Table 1). These previous renal metastasis cases underwent local treatment for lung metastasis. The minimum time from primary treatment to detection of renal metastasis was 4 years. We believe that the kidney metastasis in the present case resulted from hematogenous spread from a lung metastasis. Our patient experienced a total of 23 recurrences, including lesions located in the scapula, lungs, and kidney that were treated locally, over a period of 15 years. This suggests that repeated local treatments such as surgery and RFA for metastases may dramatically improve the 5-year survival rate of patients with metastatic chondrosarcoma. All cases of kidney metastasis were treated by surgery. Nephrectomy was performed for 2 patients and partial nephrectomy for 1 patient on review of case reports. To the best of our knowledge, the present patient in this case report is the first with invasion to the renal pelvis, requiring nephroureterectomy. Experience with additional cases will help establish better treatment strategies for the local control of renal metastasis arising from primary chondrosarcoma.

## Figures and Tables

**Table 1:** Summary of reported renal metastasis of chondrosarcoma.

Author	Year	Age	Gender	Primary location	Lung metastasis	Period from primary treatment	Treatment
Riggins and Wertlake	1965	28	F	Femur	Yes	8 years	Nephrectomy
Liguori et al.	2000	66	M	Rib	Yes	5 years	Nephrectomy
Fukumoto et al.	2013	49	F	Lumber	Yes	5 years	Partial nephrectomy
Our case	2015	55	F	Shoulder	Yes	15 years	Nephroureterectomy
